# Plasma expression of miRNA-21, − 214, −34a, and -200a in patients with persistent HPV infection and cervical lesions

**DOI:** 10.1186/s12885-019-6066-6

**Published:** 2019-10-23

**Authors:** Hongyun Wang, Dandan Zhang, Qing Chen, Ying Hong

**Affiliations:** 1 Nanjing Drum Tower Hospital, Nanjing Medical University, Nanjing, 210008 China; 2 Nanjing Drum Tower Hospital, Affiliated Hospital of Nanjing University Medical School, Nanjing, 210008 China

**Keywords:** microRNA, High-risk human papillomavirus (hr-HPV), Cervical lesions

## Abstract

**Background:**

To examine differences in the plasma levels of miRNA-21, − 214, −34a, and -200a in patients with persistent high-risk human papillomavirus (hr-HPV) infection or with cervical lesions of different grades.

**Methods:**

Venous blood was collected from 232 individuals to measure the plasma expression levels of miRNA-21, − 214, −34a, and -200a. The subjects included normal controls and patients with persistent hr-HPV infection, CIN1, CIN2, CIN3, or cervical cancer (n = 42, 31, 19, 54, 71, and 15 patients, respectively). Cervical conization specimens were collected from all the women. To ensure the accuracy of histopathology, three consecutive tissue sections with an identical diagnosis were selected, and dissection samples were taken from them for miRNA detection. Eligible cases met the inclusion criteria based on sample observation using the middle slice of sandwich tissue sections from the pathological tissue in accordance with the diagnosis of CIN1, CIN2 and CIN3 in 8, 29, and 26 cases, respectively. The miRNA-21, − 214, −34a, and -200a expression levels in the paraffin-embedded tissue samples were determined. The percentage of patients with a CIN2+ diagnosis at 30–49 years old was significantly different from that of those diagnosed with CIN1. The incidence of CIN2+ patients exposed to passive smoking was significantly different from that of CIN1- patients. The percentage of CIN2+ patients with three pregnancies was significantly different from that of those with CIN1, and the percentage of CIN2+ subjects with ≥4 pregnancies was significantly different from that of CIN1- patients. The number of CIN2+ patients with two or more induced abortions was significantly different from that of patients with CIN1. The percentage of CIN2+ patients who underwent a caesarean section was significantly different from that of patients with CIN. The percentage of CIN2+ patients with first-degree relatives with cancer was significantly different from that of those with CIN1. Among CIN2+ patients, the percentage with a first sexual encounter at ≤20 years old was significantly different from that of those with CIN1. The percentage of CIN2+ patients with ≥2 sexual partners was significantly different from that of CIN1- patients.

**Results:**

The plasma miRNA-214, −34a, and -200a expression levels were decreased in patients with more severe cervical lesions. Plasma miRNA levels in CIN1- patients were significantly different from those in CIN2+ patients. The kappa values for miRNA-21, − 214, −34a and -200a in tissue versus plasma were 0.7122, 0.9998, 0.8986 and 0.7458, respectively. The sensitivity of each biomarker for detecting CIN2 was calculated, and ROC curves of the four miRNA biomarkers were drawn. The AUC of the four plasma miRNAs was greater than 0.5, with the AUC of miRNA-21 being the largest at 0.703. The plasma miRNA expression levels exhibited at least one tie between CIN1 and CIN2. The AUCs for miRNA-21, −34a, −200a and − 214 were 0.613, 0.508, 0.615 and 0.505, respectively.

**Conclusions:**

Changes in plasma miRNA-21, − 214, −34a and -200a levels were associated with cervical lesion severity. The plasma miRNA levels in CIN1- subjects were significantly different from those in CIN2+ subjects. This analysis may help in detection of high-grade cervical lesions.

## Background

The development of cervical cancer is typically associated with high-risk human papillomavirus (hr-HPV) infection. After hr-HPV infection of the cervix, some patients become persistently infected due to environmental factors and genetic susceptibility, and a few go on to develop cervical cancer via cervical intraepithelial neoplasia (CIN) [1].

MicroRNAs (miRNAs) are non-coding, single-stranded, small RNAs between 19 and 25 nucleotides in length. miRNAs act directly on target mRNAs by annealing to their 3′ untranslated region, thereby regulating the expression of target genes. In this manner, miRNAs participate in regulating many normal cellular processes, including apoptosis, cell cycle, and methylation. In tumour cells, aberrant expression of miRNAs can promote cell proliferation, inhibit apoptosis and cellular differentiation, and direct tumour invasion and metastasis. Certain miRNA levels have also been associated with resistance to radiotherapy and chemotherapy, resulting in tumour recurrence [2]. Aberrant miRNA expression has been detected in a variety of malignant tumours, including gastric cancer, liver cancer, lung cancer, and ovarian cancer. Abnormal expression of miRNAs has also been described in some non-malignant tumours [3]. Recent studies have demonstrated that miRNAs can act as tumour suppressors or oncogenes and that the same miRNA can play opposite roles in different tumour types [4].

Previous studies have found that miRNA-21, − 214, −34a, and -200a are abnormally expressed in cervical cancer tissues and cell lines. Three of these miRNAs (miRNA-214, −34a, and -200a) function as tumour suppressors in cervical cancer [5], while miRNA-21 acts as an oncogene [6].

Several small studies of blood miRNA levels in cervical cancer have suggested that blood levels of specific miRNAs may be potential biomarkers of cervical cancer [7, 8]. Herein, we examined the relationship between miRNA levels in plasma and in corresponding cervical tissue in patients with CINs of different grades.

## Methods

### Research objective

A total of 232 subjects (see Table 1), including 190 patients admitted to the Drum Tower Hospital, Nanjing University for cervical complaints from October 2015 to January 2017, agreed to complete a questionnaire and written, informed consent for the studies. The study was approved by the clinical research ethics committee of Nanjing Drum Tower Hospital, the Affiliated Hospital of Nanjing University Medical School (22 March 2014). The patients included 31 patients with persistent hr-HPV infection (aged 42.39 ± 8.23 years), 19 with CIN1 (aged 41.93 ± 9.10 years), 54 with CIN2 (aged 42.37 ± 9.71 years), 71 with CIN3 (aged 40.77 ± 8.09 years), and 15 with cervical cancer (aged 49.73 ± 8.42 years). The 42 healthy control subjects (aged 40.94 ± 7.23 years) were negative for tuberculin and HPV and were recruited during a standard physical examination or during placement or removal of an IUD. We excluded patients with other malignancies or a history of subtotal hysterectomy or cervical cancer after radiotherapy and chemotherapy. Although none of these women smoked, some were exposed to cigarette smoke (passive smoking) every day over a period of time.

**Table 1:** Characteristics of CIN1- and CIN2+ patients

Area		Total		CIN1-		CIN2+	
		n	(%)	n	(%)	n	(%)
Total		232	(100.00)	92	(39.66)	140	(60.34)
Age	< 30 years old	21	(9.05)	9	(3.88)	12	(5.17)
	30–49 years old	158	(68.10)	60	(25.86)	98**	(42.24)
	≥ 50 years old	53	(22.85)	23	(9.91)	30	(12.93)
Passive smoking		85	(36.64)	20	(8.62)	65**	(28.02)
No. of pregnancies	≤2	123	(53.02)	55	(23.71)	68	(29.31)
	3	54	(23.28)	19	(8.19)	35*	(15.09)
	≥4	55	(23.71)	18	(7.76)	37**	(15.95)
No. of induced abortion	≤2	198	(85.34)	79	(34.05)	119*	(51.29)
	≥3	34	(14.66)	13	(5.60)	21	(9.05)
Delivery route	Cesarean section	43	(18.53)	15	(6.47)	28*	(12.07)
	Natural birth	158	(68.10)	70	(30.17)	88	(37.93)
Oral contraceptives		79	(34.05)	28	(12.07)	51	(21.98)
Benign tumors		44	(18.97)	18	(7.76)	26	(11.21)
First-degree relatives with cancer		44	(18.97)	13	(5.60)	31**	(13.36)
Sexual life	First sex ≤20 years old	46	(19.83)	11	(4.74)	35**	(15.09)
	No. of sexual partners ≥2	63	(27.16)	21	(9.05)	42**	(18.10)

In Table 1, 42 normal controls, 31 persistent HPV patients and 19 CIN1 patients were combined to obtain 92 CIN1- subjects; and 54 CIN2, 71 CIN3 and 15 cancer patients were combined for a total of 140 patients in the CIN2+ group

The percentage of patients 30–49 years old with CIN2 was significantly different from that of patients with CIN1, *p* < 0.01, χ^2^ = 13.86

The incidence of passive smoking among CIN2+ patients was significantly different from that among CIN1- patients, χ^2^ = 29.18, *p* < 0.01

The percentage of CIN2+ patients with three pregnancies was different from that of CIN1- patients, χ^2^ = 5.37, 0.01 < *p* < 0.05

The percentage of CIN2+ patients with ≥4 pregnancies was significantly different from that of those who were CIN1-, χ^2^ = 7.45, *p* < 0.01

The percentage of CIN2+ patients with ≤2 abortions was significantly different from that of those who were CIN1-, χ^2^ = 14.09, *p* < 0.01

The percentage of CIN2+ patients who had a caesarean section was significantly different from that of those who were CIN1-, χ^2^ = 4.33, 0.01 < *p* < 0.05

The percentage of patients with a first-degree relative with cancer was significantly different from that of those who were CIN1-, χ^2^ = 8.14, *p* < 0.01

The percentage of CIN2+ patients who had their first sexual encounter at ≤20 years was significantly different from that of CIN1- patients, χ^2^ = 13.91, *p* < 0.01

The percentage of CIN2+ patients who had ≥2 sexual partners was significantly different from that of CIN1- patients, χ^2^ = 8.10, *p* < 0.01

### Blood and tissue collection

Five millilitres of fasting venous blood was collected from all 232 subjects and centrifuged at 2000 g for 10 min. The upper plasma fraction was withdrawn with a pipette and stored immediately at − 80 °C. After cervical conization, formalin-fixed paraffin-embedded (FFPE) tissue samples were prepared and assessed. Cervical conization specimens were available for all the women involved in the study. To ensure the accuracy of the histopathology, three consecutive sections of the tissue with an identical diagnosis were selected, and dissection samples were taken from them for miRNA detection. Eligible cases met the inclusion criteria based on observation of FFPE samples (the middle slice of a sandwich tissue section from pathological tissue in accordance with the diagnosis). Only 63 of 190 cases were in full conformity by having such tissue specimens available. Other tissue specimens only had one or two layers consistent with the histopathological diagnosis. All samples were collected at the Department of Pathology, Drum Tower Hospital, Nanjing University.

### miRNA extraction

Plasma samples were removed from the − 80 °C freezer, thawed at room temperature for 15–45 min, and vortexed. The plasma (100 μL) was then mixed with 300 μL deionized water. Subsequent procedures were performed in a biological safety cabinet. Acid phenol, the external standard miRNA-2911, and chloroform were sequentially added to the mixture. The mixture was centrifuged at 16,000×g for 20 min at room temperature. Three layers were formed after centrifugation: miRNAs were present in the upper layer, denatured proteins in the middle layer, and chloroform in the bottom layer.

The supernatants were pooled, and isopropanol and sodium acetate were added. The mixture was allowed to stand in a − 20 °C freezer for at least 1 h and then centrifuged at 16,000×g for 20 min at 4 °C. The pellet was washed with 75% ethanol and centrifuged again. Finally, diethyl pyrocarbonate-treated water was used to dissolve the pellet for 5 min. The sample was stored at − 80 °C.

Extraction of miRNAs from FFPE samples was conducted using a miRCURY™ RNA isolation kit (EXIQON, Takara, Dalian) according to the manufacturer’s instructions. After extraction, absorbance at 260 nm was used to calculate the miRNA concentration, and the A260/A280 ratio was used to estimate the sample purity with a NanoDrop Lite spectrophotometer. The extracted miRNAs were stored at − 80 °C. Further experiments were conducted at room temperature.

### Reverse transcription and qRT-PCR

Plasma miRNAs (2 μL) were reverse transcribed into cDNA with stem loop RT primers using a reverse transcription kit (TaKaRa, Dalian). cDNA was prepared in a reaction volume of 20 μL using a TaqMan MiRNA Assay (Life Technologies, USA). The external standard, miRNA-2911, was first amplified using a 7300 qRT-PCR system (ABI, Foster, CA). Target genes and internal controls were amplified only when the difference in CT values was ≤1; otherwise, the RNA extraction was repeated. Next, miRNA-21, − 214, −34a, and -200a, as well as the internal control mixture (let7d, 7i and 7 g; see Table 2 for the primer sequences), were amplified. The PCR cycling conditions were as follows: 95 °C for 5 min, followed by 40 cycles at 95 °C for 15 s and 60 °C for 1 min. miRNAs extracted from FFPE tissue samples were subjected to the same procedures described above. U6 served as the internal control, and there was no need for the external standard miRNA-2911. Each sample was assayed in triplicate, and miRNA levels were measured three times. As in the previous experiment, miRNA-let7 was selected as the internal reference gene. All experiments were performed on ice.

**Table 2:** Primer sequences for qRT-PCR

Gene	Primer sequence
miRNA-21	5′-UAGCUUAUCAGACUGAUGUUGA − 3’
miRNA-214	5′-ACAGCAGGCACAGACAGGCAGU − 3’
miRNA-34a	5′-UGGCAGUGUCUUAGCUGGUUGU −3’
miRNA-200a	5′-UAACACUGUCUGGUAACGAUGU −3’
miRNA-2911	5′-GGCCGGGGGACGGGCUGGGA − 3’
miRNA-let7g	5′-UGAGGUAGUAGUUUGUACAGUU − 3’
miRNA-let7d	5′-AGAGGUAGUAGGUUGCAUAGUU − 3’
miRNA-let7i	5′-UGAGGUAGUAGUUUGUGCUGUU − 3’
miRNA-u6	5′-GTGCTCGCTTCGGCAGCACATATACTAAAA TTGGAACGATACAGAGAAGATTAGCATGGCCCCTGCGCAAGGATGACACGCAAATTCGTGAAGCGTTCCATATTTT −3’

### Statistical analyses

IBM® SPSS® Statistics Faculty Pack 25 was used for statistical analyses. GraphPad InStat version 5.0 was used for analysis of 2^-△CT^ values. An unpaired Student’s t-test was used to analyse differences in miRNA expression between groups, and p values less than 0.05 were considered statistically significant. The results of each miRNA plasma test were compared with those obtained from cervical tissues using a kappa test (agreement test). A kappa value ≥0.75 was interpreted as good agreement, 0.75 > kappa≥0.4 as moderate agreement, and kappa< 0.4 as poor or no agreement. We also plotted ROC curves for the different biomarkers to define the best cut-off regarding sensitivity and specificity for CIN2. The null hypothesis was the true area = 0.5.

## Results

1. There was a statistically significant difference in the rates of passive smoking between patients with persistent hr-HPV infection and patients with CIN2 and CIN3 (p < 0.05). No significant differences were noted between the groups in any other questionnaire variables (p ≥ 0.05).
2. The plasma expression of miRNA-21 increased with increasing severity of the cervical lesions (including patients with CIN1, CIN2, CIN3 and cervical cancer). The plasma levels of miRNA-21 in normal controls and patients with persistent hr-HPV infection differed significantly from the levels in patients with CIN2, CIN3 or cervical cancer (p < 0.05) but were not significantly different from levels in patients with CIN1. No significant differences were observed between miRNA-21 levels in normal controls and in patients with persistent hr-HPV infection. Patients with CIN1 did not differ significantly from those with CIN2, CIN3, or cervical cancer (Table 3). There were no significant differences in miRNA-21 expression in FFPE samples from patients with CIN1, CIN2, or CIN3 (Table 4). The results of miRNA measurements in plasma and cervix tissue were consistent, with a kappa value for consistency of 0.712.

**Table 3:** Comparison of plasma miRNA levels in patients with cervical lesions of different grades

Area	group(2^-△CT^, x ± s)							
	normal controls	persistent hr-HPV	CIN1	CIN1-	CIN2	CIN3	cancer	CIN2+
Number of cases	42	31	19	92	54	71	15	140
miRNA-21	1.96 ± 3.08	1.69 ± 2.72	3.79 ± 6.17	2.25 ± 3.93	6.88 ± 11.25	5.37 ± 6.25	6.87 ± 12.99	6.12 ± 9.37
miRNA-214	1.68 ± 3.40	2.20 ± 3.38	0.63 ± 1.79	1.63 ± 3.20	0.31 ± 1.29	0.30 ± 1.28	0.23 ± 0.31	0.29 ± 1.22
miRNA-200a	0.47 ± 0.62	0.78 ± 0.96	0.21 ± 0.35	0.52 ± 0.74	0.24 ± 0.41	0.22 ± 0.27	0.18 ± 0.19	0.22 ± 0.32
miRNA-34a	1.62 ± 2.64	2.24 ± 2.94	0.72 ± 1.39	1.64 ± 2.62	0.77 ± 1.34	0.78 ± 1.78	0.45 ± 0.27	0.74 ± 1.53

In Table 3, 42 normal controls, 31 persistent HPV patients and 19 CIN1 patients were combined to obtain 92 CIN1- subjects, and 54 CIN2, 71 CIN3 and 15 cancer patients were combined for a total of 140 patients in the CIN2+ group

Plasma miRNA levels in the CIN1- group were significantly different from those in the CIN2+ group, *p* < 0.01 (miRNA21, t = 4.34, *p* = 0.000; miRNA214, t = 4.18, *p* = 0.0001; miRNA200a, t = 3.67, *p* = 0.0002; miRNA, t = 2.98, *p* = 0.0017)

**Table 4:** Comparison of cervical tissue miRNA levels in patients with cervical lesions of different grades

Area	group(2-△CT, x ± s)		
	CIN1	CIN2	CIN3 (2-△CT, x ± s)
Number of cases	8	29	26
miRNA-21	2.91 ± 3.62	2.58 ± 9.73	3.02 ± 8.14
miRNA-214	0.05 ± 0.06	0.03 ± 0.04	0.03 ± 0.05
miRNA-200a	0.73 ± 1.35	1.60 ± 7.08	0.53 ± 1.60
miRNA-34a	0.30 ± 0.61	0.75 ± 3.14	0.16 ± 0.44
3. The plasma expression levels of miRNA-214, −200a, and -34a were decreased in patients with more severe cervical lesions (including CIN1, CIN2, CIN3 and cervical cancer). Differences in the levels of these miRNAs between normal controls and patients with CIN2 and CIN3 were significant (*p* < 0.05); however, the levels of these miRNAs in normal controls did not differ significantly from those in patients with persistent hr-HPV infection, CIN1 or cervical cancer. The plasma levels of miRNA-214, −200a, and -34a in patients with persistent hr-HPV infection were significantly different from those in patients with CIN2, CIN3, or cervical cancer (*p* < 0.05) but were not significantly different from levels in patients with CIN1. The plasma levels of miRNA-214 in patients with persistent hr-HPV infection were significantly different from those in patients with CIN2 and CIN3 (p < 0.001). However, there was no significant difference in miRNA-214 levels among patients with CIN1, CIN2, CIN3 and cervical cancer (Table 3). No significant differences were observed in the expression of miRNA-214, −200a, and -34a in FFPE samples among patients with CIN1, CIN2, and CIN3 (Table 4).
  The kappa values for mRNA-214, miRNA-34a and miRNA-200a expression in cervical tissue versus plasma were 0.9998, 0.8986 and 0.7458, respectively.
4. Receiver operating characteristic (ROC) analysis of the different biomarkers was conducted as follows. In cervical cancer screening, histologically confirmed CIN2 is usually viewed as the gold standard. We plotted ROC curves for the different biomarkers to define the best cut-off regarding sensitivity and specificity for CIN2 (Tables 5 and 6).
  Using the cut-off determined with the ROC curves, we calculated the sensitivity of the biomarkers for CIN2. We combined the 42 normal controls, 31 persistent HPV cases and 19 CIN1 cases to form 92 CIN1- cases and combined the 54 CIN2, 71 CIN3, and 15 cancer cases to form 140 CIN2+ cases. The plasma expression levels of miRNA-21 exhibited at least one tie between the CIN1- and CIN2+ patients. The AUC was 0.703 (Table 5). We reported the sensitivity of each of the biomarkers in distinguishing between CIN- and CIN2+ patients. The plasma expression levels of miRNA-21, −34a, −200a and − 214 were compared between CIN1- and CIN2+ patients. As noted above, CIN2+ patients included those with CIN2 and CIN3. The AUC values for miRNA 21, −34a, −200a and − 214 were 0.613, 0.508, 0.615 and 0.505, respectively (Table 6).

**Table 5:** Comparison of ROC Curves for Expression of miRNA in Plasma between CIN1- and CIN2+

Area Under the Curve (AUC)				
Test Result Variable (s): miRNA-21				
Area	Std. Error^a^	Asymptotic Sig.^b^	Asymptotic 95% Confidence Interval	
			Lower Bound	Upper Bound
.703	.035	.000	.634	.771

The test variable(s): plasma expression levels of miRNA-21 exhibited at least one tie between CIN1- and CIN2+ patients. The AUC was 0.703

^a^Under the nonparametric assumption

^b^Null hypothesis: true area = 0.5

**Table 6:** Comparison of ROC Curves for Expression of miRNA in Plasma between CIN1- and CIN2+

Area Under the Curve (AUC)					
Test Result Variable (s)	Area	Std. Error^a^	Asymptotic Sig.^b^	Asymptotic 95% Confidence Interval	
				Lower Bound	Upper Bound
miRNA-21	.613	.069	.113	.477	.749
miRNA-34a	.508	.067	.908	.377	.639
miRNA-200a	.615	.082	.108	.455	.775
miRNA-214	.505	.084	.941	.340	.670

The test variable(s): plasma expression levels of miRNA exhibited at least one tie between CIN1- and CIN2+ patients. The AUC values for miRNA-21, −34a, −200a and − 214 were 0.613, 0.508, 0.615 and 0.505, respectively

^a^Under the nonparametric assumption

^b^Null hypothesis: true area = 0.5

## Discussion

### Changes in miRNA-21, −214, −34a and -200a expression levels were related to cervical cancer development

MiRNA-21 promotes cell proliferation in cervical cancer cell lines and inhibits apoptosis [9]. Moreover, miRNA-34a plays an important role in tumour growth and development and in regulation of cell proliferation, differentiation, and apoptosis [10]. Interestingly, miRNA-200 is similar to miRNA-214 [11], the expression of which is decreased in patients with more severe cervical lesions, and miRNA-200 has been shown to be involved in tumour metastasis [12]. Li et al. [13] demonstrated that this miRNA is involved in epithelial-matrix transformation and destruction of cytokine receptors. In this study, we found that miRNA-34a expression was downregulated in cervical tissue from patients with more severe cervical lesions and cervical cancer compared with that in normal cervical tissue. The plasma miRNA-21, − 214, −34a and -200a levels in 232 patients and the corresponding cervical expression levels in 63 patients were analysed. We found that miRNA-21 expression was increased in patients with pathological grade lesions and that the three other miRNAs showed decreased expression in these patients. These findings are consistent with the relevant literature [7, 11, 12, 13,].

### Variation in plasma miRNA-21, miRNA-214, miRNA-34a and miRNA-200a levels may reflect different pathological changes

There have been several studies on miRNA screening in the blood of patients with cervical cancer, suggesting that blood miRNA may be a potential biomarker of cervical cancer [8]. Many studies have reported that a profile of plasma miRNA expression levels can effectively distinguish tumour patients from healthy individuals. The 10 miRNAs selected for testing in the present study were miR-21, miRNA-let7f, miRx34a, miRNA-27a, miRNA27b, miRNA29a, miR214, miRNA155, miRp200a, and miRNA199a; miRNA-let7 was used as an internal reference gene. Plasma expression changes in particular miRNAs (miR-21, miR-34a, miR-200a and miR-214) were previously shown by Xu Xiuyun [7], and their results were the basis of our study, the goal of which was to confirm that miRNA changes in plasma reflect miRNA expression changes in cervical tissues. We found that decreased plasma expression of miRNA-34a and miRNA-200a was consistent with similar expression changes in cervical lesions.

### The specificity and sensitivity of miRNA are helpful and predictive for diagnosis of cervical tumours and lesions

According to a report by Nambaru et al., in 121 cervical cancer biopsy specimens collected [14], episomal forms were more frequent in the HPV16 type, and integrated forms were more frequent in the HPV18 type (*p* = 0.011). The study found 53 miRNAs near the integration sites, 39 of which were related to cancer. The incidence of miRNAs near the HPV integration site was 78.3%, and in HPV16 type cases, the incidence was more frequent.

According to a report by Arroyo et al., miRNA circulates in the blood in a fairly stable extracellular form and has been developed as a blood biomarker for cancer and other diseases. However, the mechanism of this significant stability in the blood environment is not clear. According to the current model, cyclic miRNA is protected by membrane-bound vesicles (such as exosomes), but this has not been further studied. [15].

In a report by Allegra et al., cell data related to the role of miRNAs in the pathogenesis of various diseases are reviewed, and the latest information concerning the role of circulating miRNAs is compiled. In addition, the role of circulating miRNAs in tumour disease may be particularly important. At least 79 miRNAs are reported to be plasma or serum miRNA biomarkers of solid and blood tumours. Cyclic miRNA profiles can improve cancer diagnosis and predict the prognosis of cancer patients, while changes in circulating levels may indicate cancer susceptibility and through analysis can be used to help determine therapeutic goals [16].

According to a report by Zen et al., miRNAs, once thought to be unstable RNA molecules, are now known to be stably expressed in serum, plasma, urine, saliva and other body fluids. In addition, the unique expression patterns of miRNAs in these cycles are associated with certain human diseases, including various types of cancer. Therefore, tumour-derived miRNA levels in serum or plasma are becoming a new blood-based fingerprint to detect human cancer, especially in the early stages. [17]

In summary, precancerous lesions and cervical cancer caused by hr-HPV infection are serious diseases that threaten women’s health in our country. Alterations in the plasma expression levels of miRNA-21, − 214 m, −34a and -200a in patients with cervical lesions of different grades were coincident with similar expression changes in cervical tissue. Further studies of miRNAs and their mechanisms in related diseases should provide new ideas for diagnosis, prediction and treatment of precancerous lesions and cervical cancer.

## Conclusions

Changes in plasma miRNA-21, − 214, −34a and -200a expression levels were associated with cervical lesion severity. The plasma miRNA levels of CIN1- samples were found to be significantly different from those of CIN2+ samples. This finding may help in detection of high-grade cervical lesions.

## Data Availability

The data and material presented in this article can be obtained upon reasonable request.
